# Survival of *Brucella abortus S19* and other *Brucella* spp. in the presence of oxidative stress and within macrophages

**DOI:** 10.1007/s12223-020-00798-1

**Published:** 2020-05-28

**Authors:** Jens Jacob, Antje Finke, Martin Mielke

**Affiliations:** Robert Koch-Institute, Nordufer 20, 13353 Berlin, Germany

**Keywords:** *Brucella*, Oxidative stress, Fe^2 +^, H_2_O_2_, Macrophages

## Abstract

The evolutionary “success” of the genus *Brucella* depends on the ability to persist both in the environment as well as inside of even activated macrophages of the animal host. For that, the *Brucellae* produce catalase and superoxide dismutase to defend against oxidative stress. Since the deletion of the *mglA* gene in the *B. abortus S19* vaccine strain resulted not only in an increased tolerance to H_2_O_2_ but also in the induction of cytokines in macrophages, we here investigated the effect of oxidative stress (Fe^2+^ and H_2_O_2_) on the survival of *B. abortus S19* and the isogenic *B. abortus S 19 ∆mglA 3.14* deletion mutant in comparison with *B. neotomae 5K33*, *Brucella strain 83/13*, and *B. microti CCM4915.* These *Brucellae* belong to different phylogenetic clades and show characteristic differences in the mgl-operon. From the various *Brucellae* tested, *B. abortus S19* showed the highest susceptibility to oxidative stress and the lowest ability to survive inside of murine macrophages. *B. abortus S19 ∆mglA 3.14* as well as *B. neotomae*, which also belongs to the classical core clade of *Brucella* and lacks the regulators of the mgl-operon, presented the highest degree of tolerance to H_2_O_2_ but not in the survival in macrophages. The latter was most pronounced in case of an infection with *B. 83/13* and *B. microti CCM4915.* The various *Brucellae* investigated here demonstrate significant differences in tolerance against oxidative stress and different survival in murine macrophages, which, however, do not correlate directly.

## Introduction

Bacteria of the genus *Brucella* are members of the α-Proteobacteria. *Brucellae* are undemanding gram-negative aerobic bacteria, which produce catalase and superoxide dismutase to cope with oxidative stress (Gerhardt 1958; Plommet 1991; Moreno and Moriyon 2006).

They are phylogenetically closely related to plant pathogens and symbionts (e.g., *Agrobacterium* and *Rhizobium*) as well as to intracellular animal parasites like *Bartonella* and to opportunistic bacteria of the genus *Ochrobactrum* (Moreno and Moriyon 2006). Brucellosis is a relevant zoonosis in many regions worldwide (Cutler et al. 2005). For control, live attenuated vaccines such as *B. abortus S19* have been used for several decades but still show some substantial drawbacks (Perkins et al. 2010).

Although DNA-DNA re-association values are surprisingly high between various *Brucellae* (Verger et al. 1985), they infect various artiodactyla (e.g., sheep, goat, cattle, pigs, cetacean) as well as rodents, carnivores, primates, and even amphibians with species-related host preferences (Moreno and Moriyon 2006; Eisenberg et al. 2012) which allows the classification into several species while the genetic basis of these preferences is still unknown. Recently, three clades of *Brucella* derived from a common ancestor have been proposed: the “classical core” clade (including *B. neotomae* and *B. abortus*), the “N8” clade (including *Brucella strain 83/13*), and the “close to N8” clade (including *B. microti)* (Wattam et al. 2009, 2012, 2014).

The evolutionary “success” of the genus depends on its ability to survive both in potentially stressful environments as well as inside of even activated macrophages of the animal host (Köhler et al. 2002, 2003).

While iron is an essential factor for almost all living organisms (Pierre and Fontecave 1999; Schaible and Kaufmann 2004; Johnson and Resnick 2012), the reactive form Fe ^2+^ may also catalyze highly toxic reactions with oxygen such as the Fenton-reaction. In fact, the oxidative burst belongs to the most pronounced mechanisms of defense by infected host cells (Canning et al. 1988).

In a previous paper, we showed that an isogenic ∆*mglA* deletion mutant of *B. abortus S19* shows an increased tolerance to H_2_O_2_ and survival inside of macrophages but was found to be inhibited by Fe^2+^ in a minimal medium (Jacob et al. 2012).

To better understand the mechanisms of intracellular survival of *B. abortus* with respect to iron, a differential proteomic study was performed (Roset et al. 2017). It was shown that a low iron concentration is most likely the dominant trigger for the upregulation of proteins dedicated to reduce the concentration of reactive oxygen species, protein chaperons, and other proteins involved in detoxification (Roset et al. 2017). The authors suggested that intracellular *Brucellae* protect themselves from damage likely due to oxidative burst. Of the total number of proteins modulated by *B. abortus* in macrophages, 28% were either related to iron transport, iron storage, or iron as a cofactor.

Gene *mglA* is located in chromosome II of *B. abortus* and has recently been associated with stress responses and detoxification (Jacob et al. 2012, 2016). Close to *mglA* (a various polyols ABC transporter, ATP binding component, http://pubseed.theseed.org/?page=CompareMetabolicReconstruction&organism=430066.4), various other stress response genes are located. These include genes involved in (a) denitrification processes (including genes coding for flavo-hemoglobin structures) and (b) response to oxidative stress, e.g., BMEII0986 (BabS19_II0872) *nnrA* transcriptional regulator, *Crp/Fnr* family; BMEII0964 (BabS19_II0849) *nnrS* protein (involved in response to NO_x_).

**Fig. 1:**
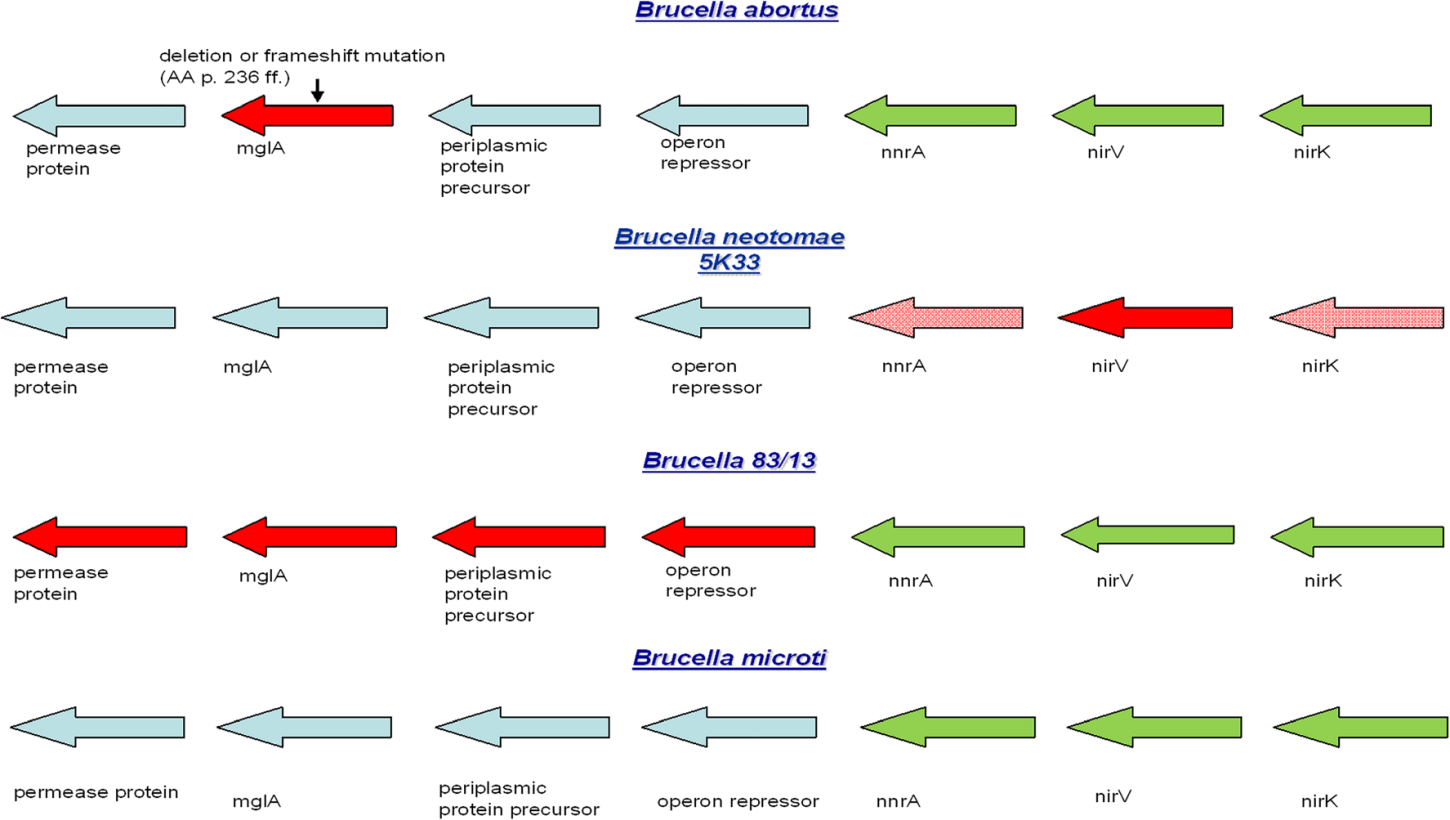
*MglA* in the context of its flanking genes (mgl-operon) in chromosome II of *B. abortus*, *B. neotomae*, *B. 83/13*, and *B. microti*. Blue arrows indicate intact genes; red arrows indicate naturally deleted complete genes. Bright red arrows indicate partly deleted genes. Green arrows indicate genes that are involved in denitrification

In order to further investigate the role of oxidative stress, Fe^2+^ and the mgl-operon (Fig. 1) in the survival of *Brucella* in macrophages, we performed a comparative study using the above mentioned members of the “classical core” clade (*B. abortus* and *B. neotomae*), the “N8” (*Brucella 83/13*), and the “close to N8” clade (*B. microti*, intact mgl-operon) of *Brucella.*

## Material and methods

### Bacterial strains

*B. abortus ∆mglA 3.14* (Jacob et al. 2012), *B. microti CCM4915* (Hubálek et al. 2008; Audic et al. 2009; Jiménez de Bagues et al. 2010), *B. abortus S19* (Crasta et al. 2008), *B. neotomae 5K33* (Stoenner and Lackmann 1957), as well as *Brucella 83/13* (Tiller et al. 2010a, b; Jiménez de Bagues et al. 2014) were grown in tryptic soy broth (TSB) and subsequently stored at − 80 °C as previously described (Hort et al. 2003). *B. neotomae 5K33* and *B. microti CCM4915* were obtained from Dr. Garin-Bastuji, B., AFFSA (Maison-Alfort, France). *B. 83/13* was obtained from Dr. Whatmore, A. (APHA Weybridge, Addlestone, Surrey, UK). *B. abortus S19* was kindly provided by Dr. Cheers, C. (Department of Microbiology, University of Melbourne, Victoria, Australia).

### Determination of survival of bacteria in various microcosms (media)

**Fig. 2:**
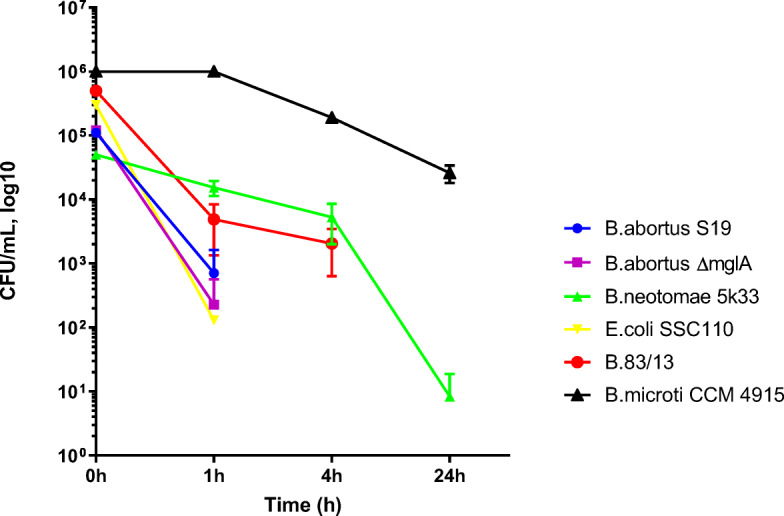
In vitro survival of various *Brucella* species in a natural Fe^2+^-containing environment (microcosm 1) as determined by CFU per mL after various times of exposition. Samples were taken at least three times and the geometric means (inclusive SD) are shown. The difference between *B. abortus S19* and *B. abortus S19 ∆mglA 3.14* at 1 h is not statistically significant

**Fig. 3:**
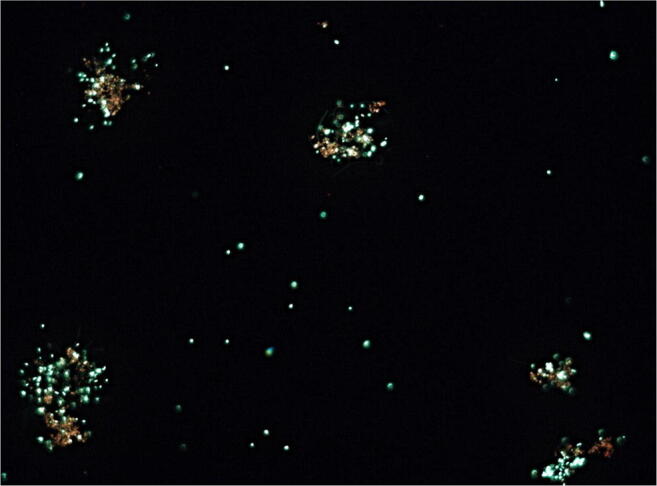
Aggregates of *B. microti CCM4915* as demonstrated by microscopy (total magnification × 400) at the end of a long-term survival experiment (18 months) in microcosm 1 at 4 °C (under anaerobic conditions) in the dark. Green fluorescence is a marker for membrane integrity

**Fig. 4:**
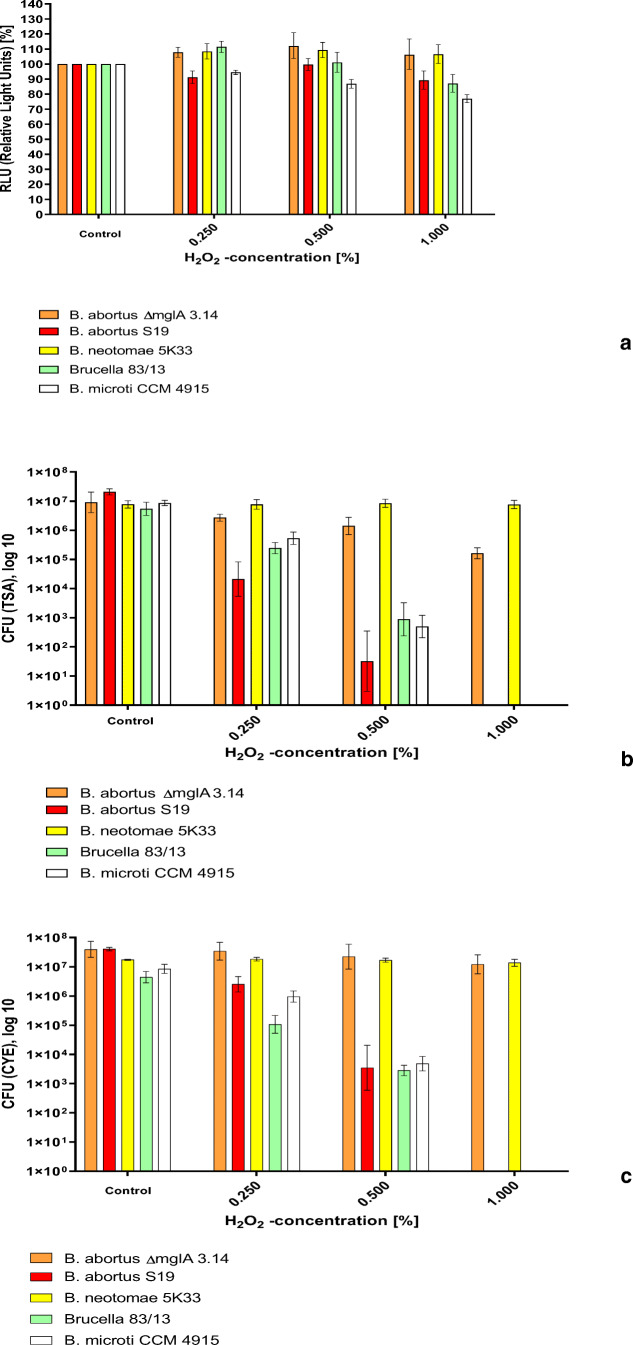
Susceptibility of *B. abortus S19A, B. abortus ∆mglA 3.14*, *B. neotomae 5K33*, *B. microti CCM 4915*, and *B. 83/13* to H_2_O_2_ in Aqua bidestillata after 30 min of exposition as determined by **a** ATP content (relative light units (RLU)), **b** CFU per mL on TSA, and **c** CFU per mL on CYE agar. Samples were taken three times and the geometric means (inclusive SD) are shown

**Fig. 5:**
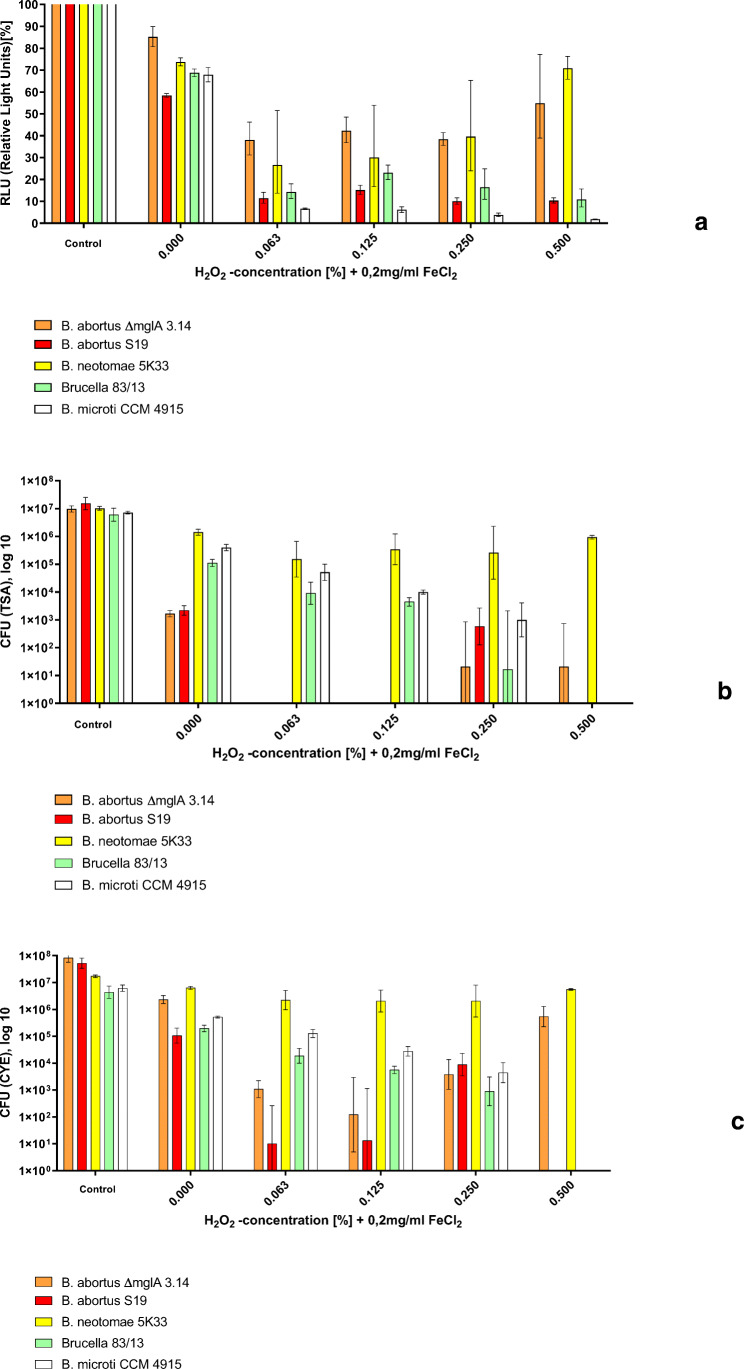
Susceptibility of *B. abortus S19A*, *B. abortus ∆mglA 3.14*, *B. neotomae 5K33*, *B. microti CCM 4915*, and *B. 83/13* to a combination of FeCl_2_ and H_2_O_2_ in Aqua bidestillata after 30 min of exposition as determined by **a** ATP content (relative light units (RLU)), **b** CFU per mL on TSA, and **c** CFU per mL on CYE agar. Samples were taken three times and the geometric means (inclusive SD) are shown

In vitro tolerance assays (results shown in Figs. 2, 3, 4, and 5) were performed in 2 media based on a natural Fe^2+^ containing water source obtained under anaerobic conditions from Saxony/Germany which we called “microcosm 1.” The natural water source microcosm 1 consisted of the following components: 11 mg/L Fe^2+^; 21. 5 mg/L Na^+^; 16.9 mg/L Mg^2+^; 19.5 mg/L Ca^2+^; 1.5 mg/L K^+^, 15. 4 mg/L SO_4_^2−^; 15.2 mg/L Cl^−^; 0.4 mg/L NO_3_^−^; 175.3 mg/L HCO_3_^−^.

Such types of natural, Fe^2+^-containing, CO_2_-rich mineral waters were scientifically well analyzed (Schulze 1995; Von Storch et al. 1996; Stumm 2002). Microcosm 1 was sampled from a Fe^2+^-containing, natural CO_2_-enriched, anaerobic cold thermal spring close to Bad Brambach (50° 16′ 00.1″ N, 12° 17′ 27.1″ E, Saxony, Germany). Geologically the source belongs to the famous group of recreational springs caused by residual volcanic activity located in West Bohemia (e.g., Karlovy Vary springs).

The spring water was aseptically sampled and anaerobically transported. It was not filtered before use but extensively tested for the absence of growth of microorganisms on CYE, TSA, and heterotrophic plate count (HPC) agar for up to 8 days at 37 °C.

Based on chemical analysis of microcosm 1 and with the goal to better standardize these environmental conditions, we created the artificial “microcosm 2.” It was designed to contain NaHCO_3_ (175 mg/L) and Fe^2+^ (NH_4_)_2_ Fe (SO_4_)_2_, (11 mg/L) in water at pH 6. The microcosm 2 was sterilized by autoclaving.

The bactericidal effect of these microcosms was caused by the oxygen influx during laboratory manipulations, i.e., the subsequent formation of oxygen radicals as result of the Fenton reaction caused by the free Fe^2+^ content.

The experiments have been performed at pH 4.5 and pH 6 which has been described for the various intracellular habitats of *Brucellae* (Fortier et al. 1995; Kulakov et al. 1997; Porte et al. 1999; Roop et al. 2004). The pH 6.0 was used for the long-term survival experiments with microcosm 1. pH 4.5 was used in microcosm 2 for the short-term luminescence TECAN assay’s measuring the intracellular ATP content.

We choose these conditions because they are similar to the intracellular conditions in the intra-cellular niche (“Brucellosome”) as it has been described as a prerequisite for the growth of *Brucella* in macrophages (Köhler et al. 2002, 2003).

The effect of FeCl_2_ and H_2_O_2_ were determined in Aqua bidestillata (pH 4.5) because of the physical-chemical nature auf the auto-phagosome, i.e., at a similar pH value.

Prior to and during the experiment, the Fe^2+^ content was checked with the Merckoquant® Iron-test (MERCK, Darmstadt, Germany, cat.nr. 1.10004.0001). This is a quantitative colorimetric test covering a range between 3 and 500 mg/L Fe^2+^. We used this test to apply the identical Fe^2+^ assay that was used for the spring water. For the experiments in the microcosms, bacterial cells from − 80 °C stocks with known and standardized viable cell counts of each *B. abortus S19, B. abortus S19 ∆mglA*, *B. neotomae 5K33*, *Brucella 83/13*, and *B. microti CCM4915* were used to inoculate Erlenmeyer flasks to yield an initial experimental inoculum of 1 × 10^6^ bacterial cells per mL in a total volume of 200 mL. The inoculated flasks were incubated at 37 °C in an anaerobic jar up to 6 days (Anaerocult A, MERCK, Darmstadt, Germany). The survival of the bacteria was determined by plating triplicate aliquots of 100 μL each on tryptic soy agar (TSA).

For long-term experiments, the inoculated microcosm 1 was kept anaerobically (Anaerocult A, MERCK, Darmstadt, Germany) in the dark at 4 °C until the tests for viability were performed.

### Determination of viability of bacteria

#### Determination of CFU/mL

For determination of colony forming units (CFU)/mL, defined volumes of bacteria were plated on tryptic soy agar (TSA) or a modified variant including charcoal (CYE). Major ingredients of CYE were 10 g ACES™ (Amino Acid Cell Rate Equine Supplement); 10 g yeast extract, 1 g α-keto-glutarate, 15 g agar, 17 g tryptone, 5 g NaCl, 3 g soy bean peptone, and 2.5 g activated charcoal.

Serial dilutions were prepared, plated, and incubated for up to 3 days at 37 °C.

#### Measurement of intracellular ATP content as indicator for bacterial viability

The bacterial intracellular ATP content, used as an indicator for the bacterial viability, was determined with a luminescence assay based on a multi-well reader Infinite 200Mpro (TECAN) and measured with the BacTiter-Glo® Microbial Viability Assay (PROMEGA, cat. nr. G8231, Madison, WI, USA). The assay procedure is based on a single reagent (BacTiter-Glo® reagent) added directly to bacterial cells in the medium and measuring luminescence.

The formulation of the BacTiter-Glo® reagent supports bacterial cell lysis and generation of a luminescent signal in a homogeneous “add, mix, measure” format. The luminescent signal is proportional to the amount of ATP present, which is directly proportional to the number of cells in culture. The BacTiter-Glo® reagent relies on the properties of a thermostable luciferase (Ultra-Glo® Recombinant Luciferase) and a proprietary formulation for extracting ATP from bacteria. This test generates a “glow-type” luminescent signal, produced by the luciferase reaction, i.e., oxygenation of luciferin is catalyzed by luciferase in the presence of Mg^2+^, ATP, and O_2_.

The experiments were performed in white 96-well microplate dishes designed for the determination of luminescence (THERMOSCIENTIFIC, cat. nr. 236105, Waltham, MA, USA). The BacTiter-Glo® reagent was used according to the manufacturer’s manual. The belonging results are shown in Figs. 4 and 5.

For this measurement of intracellular bacterial ATP under oxidative stress, the oxidative substances to be examined were spotted into the microtiter plate starting with (for example) 0, 5% H_2_O_2_; 0.2 mg/mL (1.6 mmol/L) FeCl_2_ or a combination of both and then diluted in 100 μL volumes per well. Subsequently, wells were inoculated with 10 μL of a suspension of bacteria, so that a concentration of 1 × 10^7^ bacterial cells per mL was achieved. Experiments were performed in triplicate, and a blank without inoculation of bacteria.

After 30 min of aerobic incubation 100 μL freshly prepared Bac Titre-Glo® reagent was added and the plate covered with Adhesive Film ® (NEOLAB, cat. nr.7-5170). In this step, the bacteria were lysed and ATP was measured via the luminescence-reaction using a TECAN Infinite 200 M Pro (TECAN, Gröding, Austria) multi-well reader. Results were expressed as relative light units (RLU). For that the “standard-automatic” luminescence measurement program was used.

The ascertained RLU of bacteria in Aqua bidestillata has been considered to have 100% RLU. Colony-forming units (CFU/mL) were determined in parallel. For that, separately inoculated wells were serially diluted, plated, and counted after 3 days of incubation on TSA and CYE agar. In case of the H_2_O_2_ experiments, the samples were subjected to neutralization with catalase (25 μg/mL).

#### Determination of membrane integrity as indicator for bacterial viability

In some experiments, viability of bacteria exposed to the various microcosms was also investigated for membrane integrity. The LIVE/DEAD (BacLight®) bacterial viability kit (cat. nr. L7012, MOLECULAR PROBES, Eugene, OR, USA) was used for this purpose. The test was done according to the manufacturer’s manual. Briefly, the assay works on the basis of the interaction of two fluorescing dyes, Syto 9 and propidium iodide (PI). Syto 9 is a green fluorescing dye and stains both viable and dead cells. PI is a red fluorescing dye and stains nucleoli and chromosomal structures. PI is here used for staining dead cells within a population of cells because it only penetrates cells with damaged membranes. The bacterial membrane integrity was further described as R_G/R_ ratio. This R_G/R_ ratio is a plot of the ratio of integrated green fluorescence to integrated red fluorescence (R_G/R_) versus percentage of live cells in the *Brucella* suspension.

#### Determination of bacterial membrane integrity after long-term survival in water

To test whether a status of viability but not culturability (VBNC) may exist after long-term incubation, 1 × 10^6^ CFU per mL were inoculated in 200 mL volumes of microcosm 1 and kept at 4 °C under anaerobic conditions. That was done to prevent a too rapid oxidation of Fe^2+^ and a too quick rise of oxidative stress. After 12–18 months, 100 mL volumes each of the medium were centrifuged for 15 min at 4700 rpm. The respective pellets were washed in 0. 85% NaCl, re-suspended in 10 mL NaCl and spotted in 3 × 100 μL in a microtiter plate to which 100 μL staining solution was added for the determination of membrane integrity. Five microliters of the sample was applied in case of the microscopy variant of the test.

### Macrophage infection assay

Macrophage infection assays were performed as described previously by Jacob et al. (2016). We characterized here the in vitro response of the murine splenic macrophage cell line CRL 2471(I-13.35) towards the various *Brucellae*. CRL2471 is a, non-tumor forming, adherent spleen macrophages cell line. These macrophages were previously isolated from the spleen of an adult female mouse of the LPS low responder strain C3H/HeJ by Jackson Laboratories. The macrophage growth in culture depends on colony-stimulating factor 1 (CSF1). These macrophages constitutively express CD11b/CD18 (Mac1), MHC class I, MHC class II, and colony-stimulating factor 1 receptor (CD115, CSF1R). CRL2471 is also TLR4 deficient. We finally choose CRL2471 to identify cell responses to intracellular *Brucellae* because these bacteria would be less dominated by an LPS/TLR4 interaction inside CRL2471 cells.

In general, cell line CRL2471 (Wilson et al. 1991; McCormack et al. 1992) was grown in 24-well plates with high-glucose Dulbecco’s modified Eagle’s medium (DMEM) supplemented with 10% fetal calf serum, 2 mmol/L L-glutamine and 20% CSF1 from Ladmac cells at 37 °C in humidified air containing 5% CO_2_. Cells were seeded in 24-well plates (5 × 10^5^ cells per mL). To generate Fe^2+^ saturation in macrophages, 500 μmol/L FeSO_4_ (SIGMA, Munich, Germany) was added to the DMEM cell culture medium for 12 h. Prior to the infection, this cell culture medium was replaced by standard DMEM medium.

Two hours after infection with 1 × 10^7^ bacterial cells per well each a multiplicity of infection (MOI) of 20 was obtained. Then, cells were replenished with DMEM containing 10 μg/mL streptomycine to kill extracellular bacteria. The supernatants from infected CRL2471 cell layers were plated on TSA and cultivated for up to 4 days to check for growth of putative extracellular *Brucellae*. Only assays with sterile supernatants at that point were further used for determination of viable intracellular bacteria. For that, the infected cells were 3 times washed with phosphate-buffered saline (PBS), subsequent lysed in 0.5% deoxycholic-acid, plated in appropriate dilutions on TSA agar and finally incubated at 37 °C for up to 6 days.

### RT-PCR array for the determination of mRNA coding for common cytokines

Three RT-PCR Array’s (SA BIOSCIENCES, Hilden, Germany) were used as described by Jacob et al. (2016) to measure the cellular responses of infected versus non-infected CRL2471(I-13.35) cells. The array’s used were (a) RT-PCR Common Cytokines Array (PAMM-021A, PAMM-021ZA), (b) RT-PCR Nitric Oxides Array (PAMM-062ZA), and (c) RT-PCR Dendritic and Antigen Presenting Cells Array (PAMM-406ZA). The RT-PCR Array measures bacterial mRNA transcribed in cDNA. They were used as outlined by manufacturer’s manuals.

Briefly, the RT-PCR assay was performed with RNA/cDNA obtained from macrophages grown and infected with the *Brucella* strains (refer to “Macrophage infection assay” section). Infected CRL1471 cells were then lysed by means of the RNeasy Mini Kit (QIAGEN, Hilden, Germany) to obtain total RNA. For this purpose homogenization of infected cells was done by means of a “Fast-prep®” -homogenizer (MPBIO, FP120). Briefly, lysed cells from the RNeasy Kit were transformed in Lysis Matrix M tubes (MPBIO, Eschwege, Germany, cat. nr. 6923-100) and then shaken at force 6 for 40 s. Prior cDNA synthesis RNA was further purified by the RNase-Free DNase Set (RNeasy® Mini Handbook, June 2001, pp. 99). Finally, for transcription into cDNA, the RT^2^ First Strand Kit (cat. nr. 330401, QIAGEN, Hilden, Germany) was applied.

### Statistical analysis

#### RT-PCR-Array

Statistical analysis of the RT-PCR results was performed as previously described (Jacob et al. 2016) using the data analysis portal: http://dataanalysis.sabiosciences.com/pcr/arrayanalysis.php. The results were normalized by referring to the house-keeping gene expression and shown as fold change. The fold change is described as the normalized gene expression in samples from infected versus noninfected CRL2471 cells. Geometric means from 3 independent experiments using triplicates each were used. The respective *p* values were also calculated by the data analysis tool.

#### Macrophage infection assay

Graph Pad Prism 7.04 (San Diego, USA) was used for statistical analysis (Mann–Whitney *U* test) of the data. The Mann–Whitney *U* test is a nonparametric test of the null hypothesis that it is equally likely that a randomly selected value from one group of values will be less than or greater than a randomly selected value from a second group of values. The test can be used to check whether two independent samples were selected from group of values belonging to the same distribution. Data of the macrophage infection assays were based on at least three independent experiments using triplicates each.

## Results

### Survival of *Brucella* in a natural iron-containing microcosm as determined by colony-forming units

*Brucellae* are known worldwide as a cause of zoonosis. However, they are also able to survive several days in water and can cause waterborne outbreaks (Newitt et al. 1939).

In order to investigate the effect of iron containing aquatic microcosms on the survival of *B. abortus S19*, *B. abortus S 19 ∆mglA 3.14* as well as *B. neotomae 5K33*, *Brucella 83/13*, and *B. microti CCM4915*, we exposed these bacteria to a natural iron containing water source (microcosm 1). This slightly acidic and oxidative environment demonstrated a bactericidal effect on the various *Brucella* species (Fig. 2). *B. abortus* was most susceptible. The ranking according to the survival time of the various *Brucellae* in this natural iron containing water source was *B. microti* (1 day) > *B. neotomae*/*B. 83/13* (4 h) > *B. abortus* (1 h).

This ranking was reproduced in the synthetic slightly acidic and oxidative medium microcosm 2 (data not shown).

Inspired by the results obtained for *B. microti*, we also investigated a viable but not culturable (VBNC) status by means of long-term survival experiments demonstrating that membrane integrity could be maintained over a period of 18 months for both, *B. abortus* (R_g/r_ 0,9) and *B. microti* (R _g/r_ 0,9). Moreover, ATP was still present at the end of the experiment (data not shown). In case of *B. microti* (Fig. 3), we were able to isolate single viable bacteria on TSA at the end of the experiment, while this was not possible in case of *B. abortus*.

### Effect of H_2_O_2_ on CFU/mL and intracellular ATP content

The effect of H_2_O_2_ on the various *Brucellae* is demonstrated in Fig. 4a–c. In accordance with the effect of iron in aquatic environments (Fig. 2), the various *Brucellae* could be ranked into more susceptible (*B. abortus S19*) and more tolerant *Brucellae* (*B. neotomae 5K33*, *B. microti CCM4915*, *B.83/13*) when plated on TSA*.* The *mglA* deletion mutant *B. abortus S 19 ∆mglA 3.14* demonstrated a significantly higher tolerance to H_2_O_2_ than the isogenic parental strain, comparable to the results obtained for *B. neotomae*.

Since the results obtained with the ATP assay pointed to a potential sublethal effect of H_2_O_2_ (Fig. 4a), we used a modified culture medium containing charcoal (CYE) for the recovery of the bacteria (Fig. 4c). In fact, using this medium increased the number of CFU/mL after oxidative stress especially in the case of *B. abortus.*

### Effects of a combination of Fe^2+^ and H_2_O_2_ on CFU/mL and intracellular ATP content

Since Fenton reaction (Winterbourn 1995; Kremer 2003) is known to have one of the most detrimental effects on bacteria, we performed experiments using various concentrations of H_2_O_2_ in combinations with iron (0. 2 mg/mL (1, 6 mmol/L) FeCl_2_) (Fig. 5a–c). Also, in these experiments, we observed the highest degree of tolerance in *B. neotomae 5K33.* As with H_2_O_2_ alone, the number of culturable bacteria of *B. abortus* could be increased when the charcoal containing medium was used (Fig. 5c) indicating the existence of a viable but not culturable stage.

The growth of *B. abortus S19* (which is most susceptible to oxidative stress) under conditions of various H_2_O_2_ concentrations (from 0 to 147 mmol/L, 0.5%) in the presence of constant amounts of Fe^2+^ (1.6 mmol/L) as demonstrated in Fig. 5 can be explained by the fact that the Fenton reaction follows an “S”-shaped kinetic (Liu et al. 2007). This results in an optimum of the detrimental effect depending on the relation between Fe^2+^ and H_2_O_2_ (Liu et al. 2007) and the growth of *B. abortus S19* at 0. 25% (74 mmol/L) H_2_O_2_ but not at 0.063% (18 mmol/L) or 0.125% (37 mmol/L) H_2_O_2_, respectively.

### Survival of different *Brucella* spp. in CRL2471 spleen macrophages

The results described so far point to differences in the susceptibility to oxidative stress of the various *Brucellae* tested in CRL2471 macrophages*.*

**Fig. 6:**
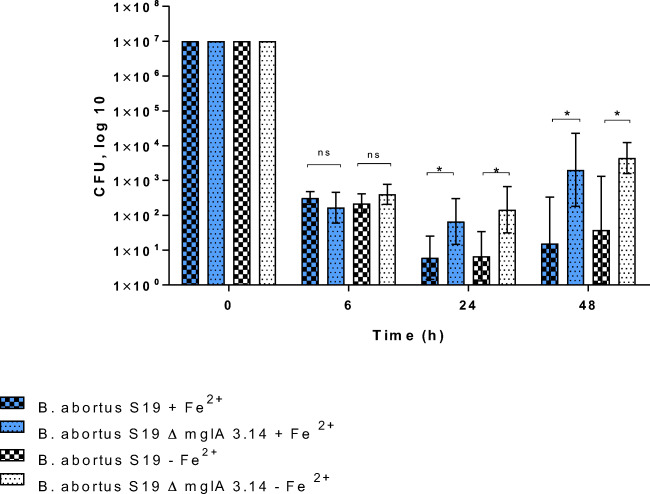
Survival of *B. abortus S19A* and the mutant strain *B. abortus ΔmglA 3.14* in CRL2471 macrophages. Cells were grown in conditioned DMEM and preincubated (or not) with 500 μmol/L (NH_4_)_2_ Fe (SO_4_)_2_·6H_2_O overnight before infection with 1 × 10^7^ CFU per mL per well. Samples were taken at least three times with triplicates each and the geometric means (inclusive SD) are shown. *(Significant) *p* 0.005

The hypothesis was that high tolerance against oxidative stress as shown most impressively for *B. neotomae* may positively correlate with the degree of growth and survival in macrophages. The results presented here extend previous findings (Jacob et al. 2012), i.e., that the *B. abortus ∆mglA* mutant strain (being more tolerant to H_2_O_2_) was found in up to 10-fold higher amounts in macrophages when compared to the parental strain *B. abortus S19* at 24 h and 48 h after infection in the presence or absence of any preincubation of the macrophages with iron (Fig. 6).

**Fig. 7:**
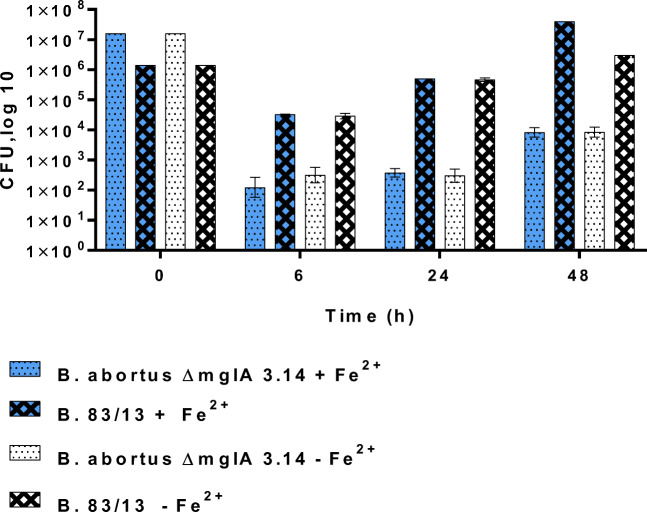
Survival of *B. abortus S19 ΔmglA 3.14* and *B. 83/13* in CRL2471 macrophages. Cells were grown in conditioned DMEM and preincubated (or not) with 500 μmol/L (NH_4_)_2_Fe(SO_4_)_2_·6H_2_O overnight before infection with 1 × 10^7^ CFU/mL per well for *B. abortus S19 ΔmglA3.14* or 1 × 10^6^ CFU/mL per well for *B. 83/13*, respectively. The differences between *B. abortus S19 ΔmglA 3.14* and *B. 83/13* at 6 h, 24 h, or 48 h are significant (*p* < 0.05)

Comparing the *B. abortus S19 ∆mglA* mutant with *Brucella 83/13* for up to 48 h revealed that infection of macrophages with the latter resulted in even higher numbers of bacteria not only at 24 h and 48 h but also at 6 h p.i. (Fig. 7).

**Fig. 8:**
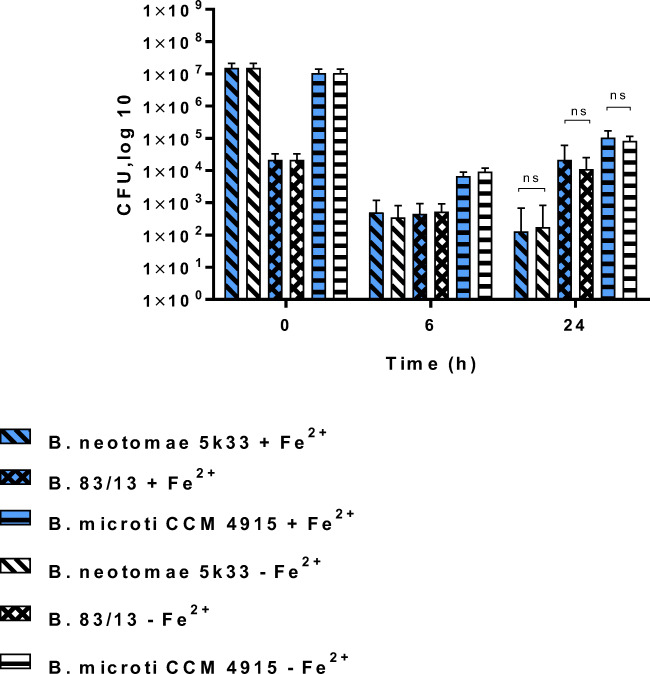
Survival of *B. neotomae 5K33, B. 83/13*, and *B. microti* in CRL2471 macrophages. Cells were grown in conditioned DMEM and preincubated (or not) with 500 μmol/L (NH_4_)_2_ Fe (SO_4_)_2_·6H_2_O overnight before infection with 1x10^7^ CFU/mL per well for *B. neotomae* and *B. microti*. In case of *Brucella 83/13*, 1 × 10^4^ bacterial cells per mL were used for infection. Samples were taken at least three times with triplicates each. ns not significant

**Fig. 9:**
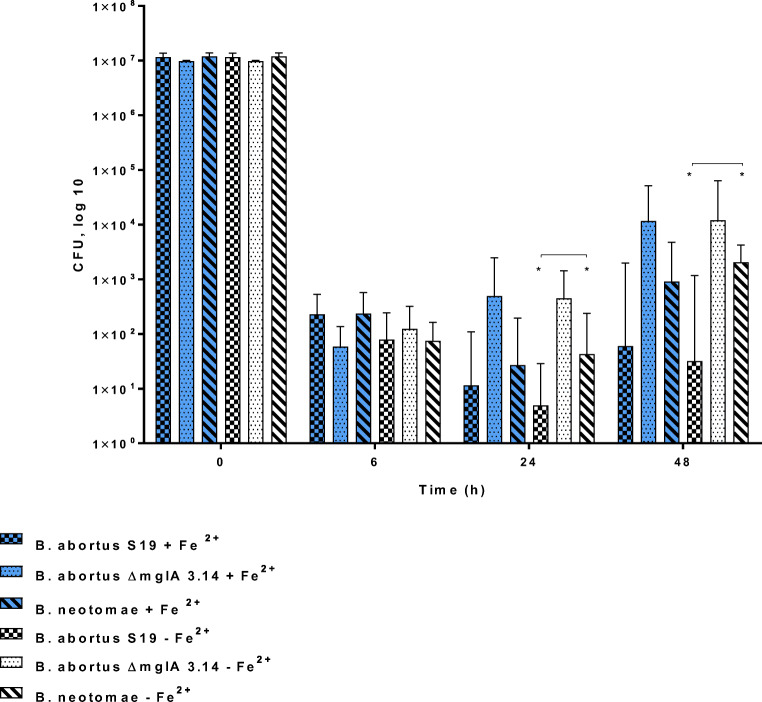
Survival of *B. abortus S19A* and *B. neotomae 5K33* in comparison to the mutant strain *B. abortus ΔmglA* in CRL2471 macrophages. Cells were grown in conditioned DMEM and preincubated (or not) with 500 μmol/L (NH_4_)_2_ Fe (SO_4_)_2_·6H_2_O overnight before infection with 1 × 10^7^ CFU/mL per well**.** Samples were taken at least three times with triplicates each. *(Significant); 24 h: *p* 0.02; 48 h: *p* 0.0005

Even when used in a significant lower infection dose (to achieve similar bacterial load at 6 h p.i.) *Brucella 83/13* was highly successful in survival and growth in macrophages, as was *B. microti CCM 4915* also (Fig. 8). The high degree of tolerance to H_2_O_2_ and Fe^2+^ observed for *B. neotomae* (Figs. 4 and 5) was partly paralleled by a comparable success in intracellular survival (Fig. 9).

### Cytokine response of CRL2471 macrophages infected with *B. neotomae 5K33*, *Brucella 83/13*, and *B. microti CCM4915*

In addition to the investigation of the behavior of *Brucellae* inside of macrophages, the cytokine response of the cells was also investigated.

The analysis of the cytokine response of CRL2471 macrophages revealed (with the exception of *B. abortus ∆mglA*) that all *Brucellae* induced a very low inflammatory response (Table 1). It is worth mentioning that *B. microti* induced significant increase of mRNA coding for IL10, IL-11, IL-17b, Fas ligand, LIF, lymphotoxin A, TNFRSF 11b, and TNFSF 13 and CD70, which was not observed in the experiments using the other *Brucellae* (Table 2).

**Table 1:** *Brucella* infection induced mRNA expression in murine CRL2471 spleen macrophages. Data were obtained by RT-PCR array 6 h after infection with *B. neotomae 5K33*, *B. microti CCM 4915*, and *B. 83/13*

Gene name	Symbol	*B. neotomae 5K33* (fold change^1^)	*B. 83/13* (fold change^1^)	*B. microti CCM 4915* (fold change^1^)
Interleukin 1α	IL1α	1.7	0.8	0.7
Interleukin 1β	IL1β	6.0 (*p* 0.009)	0.9	0.9
Interleukin 1 receptor-antagonist	IL1RN	0.6	0.8	0.1
Interleukin 6	IL6	1.2	1.2	1.7
Tumor-necrosis factor	TNF-α	8.0 (*p* 0.02)	2.7	0.2
TNF receptor 6	FAS (TNFRSF6)	1.0	0.5	2.9
Interferon β1	IFNβ1	0.7	0.8	0.8
Interleukin 12 β	IL12 β	1	2.7	1
Chemokine(CC)ligand 2	CCL2	2.0	1.3	5.3*
Chemokine (CC) ligand 17	CCL17	1.1	1.5	2.0
Chemokine (CC) ligand 3 (MIP1α)	CCL3 (MIP1α)	19.2*	7.2*	73.8*
Chemokine (CC) ligand 4 (MIP1β)	CCL4 (MIP1β)	9.5*	3.2	13.7*
Chemokine(CC) ligand5	CCL5	1.1	1.5	3.0
Chemokine(CC) ligand7	CCL7	2.7	1.7	3.1
CD 40 antigen	CD40	3.7 (*p* 0.04)	1.7	6.5*
Chemokine (CXC) ligand 1	CXCL1	2.5 (*p* 0.006)	1	3.3
Chemokine (CXC) ligand 2	CXCL2	6.5	1.3	9.,6*
Chemokine (CXC) ligand10	CXCL10	1.8	2.7	11.5*
Cxcr4	CXCR4	0.25 (*p* 0.04)	0.8	4.6*
Relb	RELB	0.8	0.7	6.6 (*p* 0.04)
Intercellular adhesion molecule 1	ICAM1	2.3	2.3	5.8*
Interferon regulatory factor 7	IRF7	1.2	2.9	6.9*
Toll like receptor 2	TLR2	3.4	1.7	10.9*
Toll like receptor 9	TLR9	1	2.3	3.5
Myc	MYC	4.1 (*p* 0.0001)	3.1 (*p* 0.006)	3.6 (*p* 0.004)
Nuclear factor kappa B	NFKB	0.8	0.7	1.7
Superoxide-dismutase	SOD2	3.5 (*p* 0.003)	1	2.4 (*p* 0.05)
Nitric oxide synthase (inducible)	NOS2	1.6	0.8	0.15

* (not significant)

^1^Fold change is the normalized gene expression in samples from infected versus non-infected CRL2471 cells. Geometric means from 3 independent experiments using triplicates each were used. The respective *p* values are given in brackets

**Table 2:** *Brucella* infection induced mRNA expression in murine CRL2471 spleen macrophages. Data were obtained by RT-PCR array 6 h after infection with *B. microti CCM 4915.* The spectrum of cytokines and chemokines demonstrated here has not been observed after infection with the other *Brucellae* investigated in this paper

Gene	*B. microti 4915* (fold change^1^)
Interleukin 10 (IL10)	16.1 (*p* 0.04)
Interleukin 11 (IL11)	5.6 (*p* 0.04)
Interleukin 17b (IL17b)	8.8 (*p* 0.04)
Fas ligand (FASL)	10.5 (*p* 0.006)
Leukemia inhibitory factor (LIF)	158.4 (*p* 0.03)
Lymphotoxin A (LTA)	6.1 (*p* 0.04)
Tumor necrosis factor receptor (TNFRSF11b)	14.0 (*p* 0.04)
Tumor necrosis factor ligand (TNFSF13)	62.6 (*p* 0.02)
Secretoglobin 3A(SCGB3A1)	392.7 (*p* 0.03)
CD 70	37.8 (*p* 0.01)

^1^Fold change is the normalized gene expression in samples from infected versus non-infected CRL2471 cells. Geometric means from 3 independent experiments using triplicates each were used. The respective *p* values are given in brackets

## Discussion

The fate of *B. abortus* inside its host is determined by the potential to survive even in activated macrophages (Moreno and Moriyon 2006). With *B. abortus S19*, an attenuated vaccine for cattle is available, which however, still has some pathogenic potential.

Until the bacteria are ingested and reach their replicative niche inside of macrophages, *Brucellae* have to cope with several harsh environmental conditions and host defense mechanisms of the infected host such as low pH, oxidative agents, or the depletion of essential factors like iron (Schaible and Kaufmann 2004; Ong et al. 2006). *Brucellae* are able to react to these challenging environmental conditions by a set of stress response mechanisms. In fact, a substantial number of genes including *mglA* are involved in stress responses, detoxification, or iron uptake (http://pubseed.theseed.org/?page=CompareMetabolicReconstruction&organism=430066.4).

In a previous paper investigating the effect of a depletion of the *mglA* gene (Jacob et al. 2006, 2012, 2016), we demonstrated that the mgl-operon may be an interesting gene region to increase safety and efficacy of the available vaccine strain *Brucella abortus S19*. The mutant was characterized by (a) decreased growth on an iron (Fe^2+^)-containing minimal basic medium (Jacob et al. 2012), (b) an increased tolerance against oxidative stress (H_2_O_2_), (c) an increased fitness in CRL2471 macrophages, and (d) a significant expression of TNFα, IL1, and IL6 cytokines in CRL2471 macrophages (Jacob et al. 2016).

The potential role of the mgl-operon is further highlighted by (a) iron-dependent downregulation of BAB2_0938 (ABC-type transport system, directly beside of *mglA*) in macrophages as recently found by Roset et al. (2017), (b) the expression of the analogue gene BMEII0983 as described by Eschenbrenner et al. (2002), and (c) the directly flanking genes *nirK*, *nirV*, and *nnrA* which were previously assessed by Baek et al. (2004) in *B. neotomae.*

The bacteria investigated in this paper show substantial differences in this region (Fig. 1).

*B. microti*, *B. 83/13*, and *B. neotomae* are among a group of *Brucella* species which were originally isolated from mammalian species of the order rodent including mice (Stoenner and Lackmann 1957; Baek et al. 2004; Audic et al. 2009; Covert et al. 2009; Tiller et al. 2010a, b; Moustafa et al. 2010, 2011).

Based on comparative genomics, the *Brucellae* used in this paper can be sub-grouped into (a) the “classical core clade” *Brucella* clade (*B. abortus*; *B. neotomae 5K33*), (b) the “N8” clade (*Brucella 83/13*) and c) the “close to the N8” clade (*B. microti CCM4915*) according to Wattam et al. (2009, 2012, 2014). While *B. abortus* is typically isolated from cattle, the other *Brucellae* have rodent reservoirs and are phylogenetically older branches of the genus *Brucella*. *B. microti* has been isolated from *Microtus arvalis* (Hubálek et al. 2008; Audic et al. 2009). *Brucella 83/13* has been isolated from rodents in Australia (Tiller et al. 2010a, b) and *Brucella neotomae* from *Neotoma lepida* (Stoenner and Lackmann 1957). Only in *Brucella 83/13* as well as in *Brucella inopinata* (Tiller et al. 2010a, b) the mgl-operon is absent from the *Brucella* genome. In all other *Brucellae*, it is present. We therefore decided to include *B. 83/13* in the study presented here.

In this investigation, we exposed the various *Brucellae* to Fe^2+^ and H_2_O_2_ both in the microcosm experiments (Figs. 2 and 3) as well as in the short-term survival assays (Figs. 4 and 5).

A role of iron in bacterial growth and innate resistance of macrophages is widely accepted (Johnson and Resnick 2012). Fe^2+^ has been shown to be an essential factor for intracellular bacteria to survive inside their host cells (Schaible and Kaufmann 2004). In addition, it has recently been shown that 28% of the number of intracellularly modulated proteins of *B. abortus* are related to iron transport, iron storage, iron as a cofactor, or the iron regulator *Irr* (Roset et al. 2017). The sources of iron (reactive Fe^2+^, non-reactive Fe^3+^, haem or haem-containing proteins) and the metabolic needs for these ions differ depending on the respective microenvironment, e.g., water or the replicative niche inside of macrophages (Köhler et al. 2002, 2003).

The effect of iron on the fate of bacterial growth is the result of a complex homeostasis between its role as an essential growth factor on the one side and its toxic effects caused by Fe^2+^ especially in combination with oxygen (e.g., Fenton reaction or Haber-Weiss reaction), on the other side (Repine et al. 1981; Crichton et al. 2002; Theurl et al. 2005).

In fact, a role of the iron equilibrium and various oxidative mechanisms on the intracellular growth of *B. abortus* has been discussed by Baldwin et al. (1993). These authors concluded that macrophages can be stimulated to increase their ability to control the growth of opsonized *Brucella* through IFNγ and the generation of reactive oxygen intermediates. They demonstrated that macrophages activated with IFNγ and supplemented with Fe^2+^ are very effective in killing intracellular *B. abortus* through the generation of hydroxyl radicals and other oxidative intermediates (Baldwin et al. 1993). They suggested that it might be of interest to determine whether molecules involved in Fe^2+^ transport may be of relevance for intracellular survival.

From the bacteria investigated here, *B. abortus S19* (the currently used vaccine strain of *B. abortus*) showed the highest susceptibility to oxidative stress induced by Fe^2+^, H_2_O_2_, or a combination of both (Fenton reaction) in three different readout assays (CFU/mL, ATP level and membrane integrity). The highest tolerance was found in *B. neotomae*, the strain missing the whole mgl-operon control region (Fig. 1), followed by *B. abortus ∆mglA 3.14* and *B. microti* (Figs. 4 and 5). In fact, these strains survived best in a milieu containing a combination of H_2_O_2_ and FeCl_2_ (Fig. 5). This behavior was paralleled by long-term survival in an iron-containing natural environment (microcosm 1) as determined by culture, membrane integrity, and the presence of low levels of ATP (Fig. 3).

In case of *B. neotomae*, the tolerance to oxidative stress may be explained by the ability to produce high amounts of catalase (Harmon and Adams 1987; Bricker et al. 1990; Moreno and Moriyon 2006). However, as exemplified by the isogenic deletion mutant of *B. abortus S19* (*B. abortus ∆mglA3.14*), the *mglA* gene seems also to be involved in tolerance to H_2_O_2_ (Fig. 4).

The results obtained in the short-term in vitro assays (Figs. 4 and 5) are also compatible with the results from the long-term experiments in water (Figs. 2 and 3), i.e., *B. microti* demonstrated long-term survival.

In an attempt to correlate the findings obtained in the various environmental microcosms with the survival of the bacteria inside of macrophages, we used a formerly described macrophage assay in the presence or absence of any preincubation of the macrophages with iron (Jacob et al. 2016). The hypothesis was that increased tolerance to oxidative stress may positively correlate with the survival of these bacteria in macrophages.

In these experiments, using TLR4-deficient CRL2471 macrophages and nonopsonized bacteria in the absence of IFNγ, *Brucella 83/13* (Figs. 7 and 8) and *B. microti* (Fig. 8) demonstrated a pronounced significant multiplication in infected macrophages when compared to *B. abortus S 19* and *B. neotomae.*

The infection of macrophages and the intracellular survival of *Brucella* had been divided into several phases starting with the phagocytic uptake of the bacteria (Porte et al. 1999; Köhler et al. 2002, 2003; Roop et al. 2004).

During the first 2 h after infection of macrophages, the *Brucella* localize within single phagosomes, without signs of microbial degradation. The number of ingested intracellular *Brucella* depends upon the size of the inoculum and the presence or absence of antibodies. Fusions between lysosome-like granules and some *Brucellae* containing phagosomes are evident at these early times.

Within 12–15 h after ingestion, the amount of *Brucellae* decreases significantly. Microscopic investigations revealed that a part of *Brucella*-containing compartments have fused with lysosomes (pH 4.5–4.8) and many of the *Brucellae* had been found degraded.

Evasion of the oxidative burst by *Brucellae* has been discussed as a way for *Brucella* to adapt to the host cell especially under opsonized conditions (Harmon and Adams 1987; Canning et al. 1988; Köhler et al. 2002, 2003; Moreno and Moriyon 2006).

Between 15 and 48 h postinfection, the number of virulent intracellular bacteria typically increases and macrophages become an adequate niche for replication. The number of intracellular *Brucellae* per cell usually increases until the cytoplasm of the phagocytic cells is filled with bacteria. Finally, in vitro-infected cells rupture and bacteria are released.

Beside the results for *B. abortus* and *B. neotomae*, published data obtained in bone marrow-derived macrophage survival assays are available for *Brucella strain 83-210* (Jiménez de Bagues et al. 2014) as well as for *B. microti* (Jiménez de Bagues et al. 2010; Ouahrani-Bettache et al. 2019).

In our experiments using CRL2471 cells, infection of macrophages took place in the absence of specific antibodies and TLR4. In a previous paper (Jacob et al. 2016), we have shown that *B. abortus* was able to penetrate and survive in these cells even after stimulation by IFNγ which resulted in the intense induction of inflammatory cytokines as well as SOD and NOS2.

In the experiments presented here, *B. microti CCM4915* and *Brucella 83/13* showed higher numbers of viable bacteria at 6 h p.i. in CRL2471 macrophage cells when compared to *B. abortus* including the *B. abortus ΔmglA* deletion mutant and *B. neotomae* in the presence or absence of any preincubation with Fe^2+^. All investigated bacteria increased in numbers within 24 h and 48 h after infection but to differential degrees (*Brucella 83/13* > *B. microti* > *B. neotomae/B. abortus S19*) irrespective of the preincubation with Fe^2+^ (Jiang and Baldwin 1993; Baldwin et al. 1993), while *B. neotomae* demonstrated a pronounced tolerance to oxidative stress, most probably due to its capacity to produce catalase; this was not reflected by a comparable degree of increased survival in macrophages (Fig. 9).

In addition to the survival in macrophages, we investigated the cytokine response of the cells to the infection. In a previous paper, we demonstrated the inflammatory response of CRL2471 cells to *B. abortus S19* and the *B. abortus ∆mglA* mutant (Jacob et al. 2012). This cell line still depends on the presence of colony-stimulating factor 1 (CSF1) and is derived from LPS-resistant (TLR4-deficient) C3H/HeJ mice. While numbers of viable bacteria did not differ significantly between the vaccine strain and the deletion mutant at 6 h postinfection, a higher bacterial load was measured in case of the mutant at 24 h and 48 h after infection. This was also true when IFNγ was used for macrophage activation. A comprehensive gene expression profile of macrophages revealed that the mutant strain *B. abortus ∆mglA 3.14* elicited a stronger cellular response of the splenic macrophages as compared to the parental vaccine strain. This was most prominent for the pro-inflammatory cytokines IL 1, TNFα, and IL6 as well as for the chemokine ligands CXCL1, CXCL2, CXCL10, CCL2, CCL5, CCL7, CCL17, and the co-stimulatory molecules CD40 and ICAM1. The addition of IFNγ after infection not only resulted in a dramatic increase of the translation of the before mentioned genes but also in the translation of IFNß1, IL12ß, MIP1 and CCL3, CCL4, NOS2 and SOD2, and FAS but, nevertheless, did not prevent multiplication of the bacteria.

In this paper, we extended these data about the cytokine response of CRL2471 macrophages to *B. neotomae*, *Brucella 83/13*, and *B. microti.* While the very low inflammatory response to *B. neotomae*, *Brucella 83/13*, and *B. microti* did not significantly differ from the response described for *B. abortus S19*, the cellular response to *B. microti* was characterized by a prominent transcriptional response of mRNA coding for IL 10, IL 11, IL 17b, FASL, LIF, LTA, TNFRSF 11b, TNFSF13, secretoglobin, and CD70 which has not been demonstrated for *B. abortus*, *B. neotomae*, and *Brucella 83/13*. In this context, it is worth mentioning that *B. microti* has been shown to cause lethal infections in rodents (Hubálek et al. 2008; Audic et al. 2009).

In conclusion, *Brucellae* can be grouped into the “classical core clade,” the “N8,” and “close to the N8” clade (Wattam et al. 2009, 2012, 2014). In addition, the various species show characteristic differences in the mgl-operon (Fig. 1). Recent findings about the function of *mglA* point to a role as an ABC-type transporter potentially involved in stress response and detoxification*.*

We therefore performed a broader comparative study investigating the behavior of *B. abortus S19*, *B. abortus ∆mglA*, *B. neotomae*, *B. microti*, and *B. 83/13* with respect to their tolerance to oxidative stress (Fe ^2+^, H_2_O_2_) and survival inside of macrophages. From the bacteria tested, *B. neotomae,* missing the whole mgl-operon control region and producing higher amounts of catalase, demonstrated the highest tolerance to even combinations of Fe^2+^ and H_2_O_2_ (Fenton reaction).

Although the deletion mutant *B. abortus ∆ mglA 3.14* demonstrated increased tolerances to oxidative stress and survival inside of infected TLR-4 deficient macrophages, the results obtained with *B. neotomae*, the most tolerant strain, pointed to the fact that there was no simple association between survival in cells and tolerance to oxidative stress in vitro*.* This was underlined by the findings obtained with the other *Brucellae. B. 83/13* presented a pronounced uptake into macrophages and *B. microti* induced a type of cytokine response, which was not observed with the other *Brucellae.*

Therefore, although *Brucellae* show a very high degree of genetic similarity the bacterial burden of macrophages infected with *Brucellae* seems to be determined by various factors including not only oxidative stress response but also several other, so far not well-described factors.
